# A REVIEW ON EMERGING PATHOGENESIS OF COVID-19 AND POINTS OF CONCERN FOR RESEARCH COMMUNITIES IN NIGERIA

**DOI:** 10.21010/ajid.v15i2.7

**Published:** 2021-03-18

**Authors:** Mubarak Muhammad, Salisu Ahmed Ibrahim, Isyaku Umar Yarube, Bashir Bello

**Affiliations:** 1 Department of Physiology, College of Medicine, University of Ibadan, Nigeria; 2 Department of Human Physiology, College of Health Sciences, Bayero University Kano, Nigeria; 3 Department of Physiotherapy, College of Health Sciences, Bayero University Kano, Nigeria

**Keywords:** COVID-19, SARS-CoV-2, Coronavirus, Pandemic, Pathogenesis

## Abstract

**Background:**

COVID-19 remains an emerging pandemic that continuously poses an alarming threat and challenge to economic, social and well-being of the people throughout the world. It also remains an evolving disease which complete pathogenesis that translates into clinical features is only just emerging by each second of the day. There have been observations about the emerging trends of the disease in Nigeria like in any other country in the world where there is outbreak. This study examined from evidence-based literature the emerging pathogenesis of COVID-19 and important points of concern of the disease in Nigeria.

**Materials and Methods:**

The paper reviewed published articles in PubMed and Google Scholar using search terms ‘COVID-19” and “SARS-CoV-2”, as well as searched for general COVID-19 information on internet.

**Results:**

The result summarized literature on emerging pathogenesis of COVID-19 and important points of concern as well as research questions as to the peculiar trends of the disease in Nigeria.

**Conclusion:**

Pathogenesis of COVID-19 remains an emerging knowledge and there are many important research questions that need to be scientifically answered for a successful containment of COVID-19 in Nigeria. It is recommended that all members of intellectual research communities should join the fight against COVID-19 pandemic.

## Introduction

Coronavirus disease 2019 (COVID-19) remains a novel and ongoing evolving pandemic that poses unprecedented enormous threat and challenge to the economic, social, and health of humanity all over the regions of the world, although with approved vaccines currently available but specific definitive therapy still remain elusive (Zheng, 2020). The economic constrain of COVID-19 pandemic on global scale according to the preliminary report by United Nations Conference on Trade and Development Agency (UNCTAD) was 2 trillion Dollar as of 9 March 2020, it was estimated that it could reach a projection of 10 trillion Dollar which is about one-eighth of global growth domestic product (GDP) with a palpable potential global economic recession threat (Reuters, 2020). The social impact of the disease is massive as it was reported to have directly grounded social activities of half of humanity (The New York Times, 2020). COVID-19 has affected 105,429,382 people worldwide as of 7^th^ February 2021, out of which is a total of 2,302,614 confirmed deaths in more than 215 countries, areas, or territories of the world (WHO, 2020a). As a result, the disease has stimulated wide fields of research and study into the search for potential therapy or prevention, and currently there are more than 500 registered clinical trials in the database of World Health Organization’s International Clinical Trials Registry Platform (WHO ICTRP) that intended to produce potential definitive therapy against COVID-19.

The important landmark development into the disease outbreak started with the rising source of concern for an unexplained pneumonia of unknown etiology among patients who were employees of the Huanan seafood wholesale market in Wuhan, Hubei Province, China in December, 2019. (Wang *et al.*, 2020). A new coronavirus was subsequently identified, isolated and reported on 31^st^ December 2019 from the respiratory epithelium of the patients, and was tentatively named initially by China’s National Health Commission as “2019-novel coronavirus” (2019-nCoV) on 7^th^ January 2020 with the term novel denoting that the virus is only emerging and requires further extensive studies. On 11^th^ February, 2020, the new coronavirus was officially renamed from 2019-nCoV to severe acute respiratory syndrome coronavirus 2 (SARS-CoV-2) by Coronavirus Study Group (CSG) of International Committee on Taxonomy of Viruses (ICTV), and on the same date the World Health Organization (WHO) formally termed the disease caused by SARS-CoV-2 as “coronavirus disease 2019”. WHO publicly stated on 30^th^ January 2020 that the Chinese outbreak of COVID-19 constitutes a Public Health Emergency of International Concern (PHEIC), and following an exponential growth curve in the worldwide stretch of the virus, the WHO classified COVID-19 as pandemic on 11^th^ March 2020 to signify a worldwide extension in the tidal wave of the SARS-CoV-2.

Since the index case of COVID-19 in Nigeria was first confirmed on 27^th^ February 2020 involving an Italian citizen that returned from Europe, by 5^th^ May 2020, Nigeria was into the week 10 of the disease outbreak. By that date, the Nigeria Centre for Disease Control (NCDC) has reported 2,950 confirmed cases of COVID-19, 481 cases discharged and 98 deaths in Nigeria (NCDC, 2020). There have been concerns and observations about the trends of COVID-19 in Nigeria and other African countries relative to other regions of the world (Lone and Ahmad, 2020). The first confirmed case of COVID-19 in the United State (US), the current global epicenter of the disease was on January 20, 2020, but in 10 weeks into the period of outbreak (30^th^ March 2020) the number of confirmed cases in US according Johns Hopkins University data was 161,807 cases and 2,978 deaths. Similarly, the number of confirmed cases moved to 143,626 cases and 18,279 deaths from the first index case (31^st^ January 2020) within 10 weeks in Italy, and in Iran, moved from first index case (19^th^ February 2020) to 91,472 confirmed cases and 5,806 within 10 weeks.

Currently, concerted efforts have been intensified all over the globe with scientists working with unprecedented work-rate to understand SARS-CoV-2 and develop targeted therapeutics (Sun *et al.*, 2020). Understanding the emerging knowledge on the pathogenic mechanism of the disease is critical for potential therapeutic strategies against COVID-19.

This paper therefore, discussed emerging pathogenesis of COVID-19 infection and important points of concern as well as research questions pertaining to the peculiar trends of the disease in Nigeria.

## Materials and Methods

This study reviewed literature using PubMed and Google Scholar search engines and search strategy were conducted using keywords for coronavirus (as “COVID-19” OR “CoV” OR “Coronavirus”) and SARS-CoV-2 to yield published articles written in English language. This study also considered general COVID-19 information available on internet that were presented with URL sources.

### Structural biology of SARS-CoV-2

Coronaviruses (CoVs) are the largest family of positive-strand RNA viruses that varied from 60-140 nm diameters and cause respiratory illness in both humans and other vertebrate animals. The word corona was coined from Latin word for ‘crown’ owing to its outward surface show of spike like projections that looks like crown shape when viewed under electron microscope (Chavez *et al.*, 2020). Before COVID-19 pandemic, there were two previously zoonotic coronavirus epidemics of lower respiratory infections; the severe acute respiratory syndrome (SARS) in 2002, and the Middle East respiratory syndrome (MERS) in 2012.

The taxonomy of CoVs is classified in order *Nidovirales*, family *Coronaviridae*, subfamily *Orthocoronaviridae*, and this subfamily has about four subdivisions of genera; the alpha (α)-coronavirus, the beta (β)-coronavirus, the gamma (γ)-coronavirus and delta (δ)-coronavirus. The γ and δ coronaviruses have been known to infect birds, while the α and β coronaviruses have been the genera known to infect mammals and the identified coronaviruses that affects humans such as SARS-CoV, MERS-CoV and SARS-CoV-2 all belongs to the genus of β-CoVs. With the discovery and surfacing of SARS-CoV-2, there are currently at least seven known coronavirus species described to date as being responsible for respiratory infection in humans: HCoV-229E and HCoV-NL63 belonging to the genus α-coronavirus; and HCoV-OC43, HCoV-HKU1, MERS-CoV, SARS-CoV, and SARS-CoV-2 which belong to the genus β-coronavirus (Devaux *et al.*, 2020).

The genomic structure of SARS-CoV-2 was critically observed and revealed that SARS-CoV-2 is an enveloped positive single-stranded RNA (ssRNA) virus with RNA genome of 30 kb (Aggarwal *et al.*, 2020). The viral genome structure consists of two parts; the part that encodes range of 6 to 11 varied number of open reading frames (ORFs), and the part that encodes essential structural proteins. Within the ORFs part; the first open reading frame (OFR 1a/b) constitutes the largest gene in SARS-CoV-2 as it contains two-third of the viral RNA that encodes two polyproteins which are polyprotein 1a (pp1a) and polyprotein 1b (pp1ab), and 16 non-structure proteins (NSPs) from NSP1-NSP16; the remaining ORFs part SARS-CoV-2 constitutes the remaining one-third of the genome that encodes four essential structural proteins such as spike (S) protein, envelope (E) protein, membrane/matrix (M) protein, and nucleocapsid (N) protein, as well as several accessory and other structural proteins with unknown functions which do not participate in viral replication (Shereen *et al.*, 2020).

### Emerging pathogenesis of COVID-19

COVID-19 remains currently an emerging disease whose complete pathogenesis is still evolving with so many knowledge gaps that still need to be filled over the course of time. From the available knowledge in the literature, there are basically three pathophysiological steps that are now identified to be the hallmark of COVID-19 pathogenesis namely; viral replication, immunopathogenesis and cytokine storm (Chatterjee *et al.*, 2020).

The SARS-CoV-2 viral replication step begins as the virus uses its surface protein S most frequently called “spike protein” or “spike glycoprotein” to recognize and bind with the host transmembrane cellular receptor protein which was identified as angiotensin-converting enzyme 2 (ACE2) receptor found in the mucous membrane of human lower respiratory tract (Walls *et al.*, 2020). This causes virus-membrane fusion that allows the viral genome RNA (gRNA) to release into the cytoplasm to replicate or make multiple copies of itself via the following steps: translation of viral gRNA to form the two polyproteins which encodes NSPs, and create replication-transcription complex (RTC) in double-membrane vesicle; proteolysis of the translated polyproteins with viral 3C–like proteinase; replication of viral RTC to continuously form subgenomic RNA that encode accessory and structural proteins including essential structural proteins; assembly of viral components such as the newly formed subgenomic RNA and the four essential structural proteins into viral particle buds; release of the replicated virus out of the cells by binding and extrusion of the virion-containing vesicles with the plasma membrane.

Immune response toward the invading SARS-CoV-2 typically follows a double-edged sword fashion; a precise response in some patients’ immunity signifies regulation and resolution of the virus; whereas out of control immune response in some subset of patients result in immunopathogenesis that signifies over-reacting, dysfunctional and out of control immune response which consequently lead to cytokine storm syndrome. Optimal increase in immune reaction that goes with optimal increase in production of inflammatory cytokines is a delicate balance required to regulate and eliminate SARS-CoV-2. However, over-activation of the immune system leading to hyperinflammation creates clinical characteristics associated with negative outcome such as severe symptoms of COVID-19 and mortality (Chen *et al.*, 2020).

The innate immune response constituted first line of defense against SARS-CoV-2 invasion in the airways (Ye *et al.*, 2020), and this immunity composed of epithelial cells, alveolar macrophages and dendritic cells which fights against the virus till adaptive immunity is involved. At optimal and ideal state immune system, the ACE2 expressed respiratory invaded epithelial cells of the host innate immunity is initiated by virus-cell interactions where the viral RNA becomes recognized. Recognition induces recruitment of viral adaptor proteins such as TIR-domain-containing adaptor protein (TIRAP) including TIR-domain-containing adapter-inducing interferon-β (TRIF), mitochondrial antiviral-signaling protein (MAVS) and stimulator of interferon genes protein (STING). These adaptor proteins further activate a series of cellular cascading events that forms transcription of various pro-inflammatory genes such as nuclear factor kappa-light-chain enhancer of activated B cells also known as nuclear factor kappa B (NFκB) and interferon regulatory factor 3 (IRF3) that together ultimately generates immune mediators that leads to control and resolution of the invading virus. How SARS-COV-2 progress to subverts and overcome innate immune response remains largely elusive, but it has been illustrated in SARS-CoV that nucleocapsid (N) protein helps the virus escape from such immune responses, as such further activating adaptive immune response of both cellular and humoral immunity to respond against the virus. The combined immune response of innate and adaptive immunity which later evolved upon antigen presentation plays a critical protective role against SARS-CoV-2 (Tay *et al.*, 2020).

However, if these series of processes initiated by virus-cell interactions and appropriate innate immune response goes otherwise inappropriate and the coordinated immune response becomes impaired, immunopathogenesis ensues to cause dysfunctional immune response that results in physiological alteration of pulmonary function such as: release of the replicated virus from the ACE2 expressed epithelial cell linings of the alveoli that cause mechanical irritations of nerve endings to initiate cough reflex and produce dry cough symptom; release of inflammatory mediators by the infected and damaged epithelial cells produce local inflammation that leads to increased permeability of the alveoli and accumulation of fluids into the alveoli that typically results in alteration of pulmonary gas exchange, difficulty in breathing symptom, hypoxemia or fall in saturated partial pressure of oxygen (SPO_2_) (Rockx *et al.*, 2020). The local inflammation establishes pro-inflammatory feedback loop that combine with decrease in surfactants due to damaged type II pneumocytes and fibrosis of lung tissue to continuously creates and amplify the pathophysiological processes that damage lung structure and reflects the respiratory complications of COVID-19 (Mason, 2020).

Cytokine storm (CS) is systemic inflammatory response resulting in excessive and uncontrolled release of pro-inflammatory cytokines, and cytokine storm syndrome is a disease feature in COVID-19 that correlates with severity of the infection and therefore marks the critical stage in clinical progression of COVID-19 to severe pneumonia, acute respiratory distress syndrome (ARDS), respiratory failure and death, as well as COVID-19 manifestations of multiple-organ dysfunction syndrome and multi-organ failure (Tan and Aboulhosn, 2020). Cytokines are low-molecular-weight proteins formed by immune cells as well as stromal cells in response to arrival appropriate production stimulus to control a number of physiological and pathological processes including innate and acquired immune responses to pathogens, and a plethora of inflammation (Muhammad, 2019). Cytokines are classified into six groups as: interleukin (IL), interferon (IFN), tumor necrosis factor (TNF), colony stimulating factor (CSF), chemokines and growth factor (GF). Some of the cytokines found to be abnormally elevated among COVID-19 patients includes pro-inflammatory interleukins, interferon gamma (IFN-γ), tumor necrosis factor alpha (TNF-α), macrophage colony-stimulating factor (MCSF), monocyte chemoattractant protein 1 (MCP-1), and hepatocyte growth factor (HGF).

**Figure 1:**
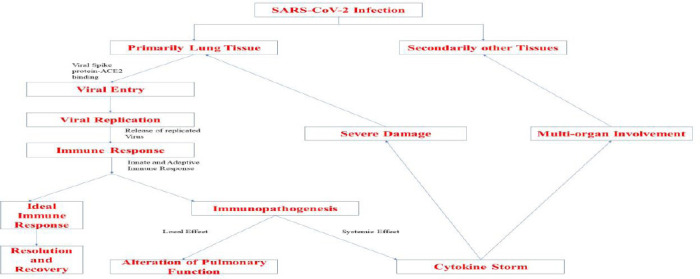
Schematic representation illutrating pathogenesis of COVID-19

There are emerging indications relating COVID-19 beyond development of respiratory illness to manifestations of neurological, gastrointestinal, nephrological, hepatobiliary, and cardiovascular illnesses. Although the rationale behind these multi-organ involvement remains an area that needs extensive study, cytokine storm has been preliminarily implicated as most likely pathophysiological mechanism. For instance, Cytokine storm can result in sepsis due to cytokine storm related systemic dysregulation of host response to infection, sepsis in turn is associated with hypercoagulable and hyperthrombotic body state. Hypercoagulability and hyperthrombotic state that causes microthrombi obstruction of cerebral microvasculature have been recently implicated as the cause of neurological manifestation if ischemic stroke due to COVID-19 (Divani *et al.*, 2020). Neurological manifestation of COVID-19 has become increasingly conspicuous with accumulating level of evidence implying that neurological symptom such as dysfunction of smell and taste might precede the much talk-about respiratory symptoms (Koralnik and Tyler, 2020). Emerging evidence on mechanism of multi-organs involvement proposed that the neurological manifestation can be attributed to both direct factor of neuroinvasion into the CNS to infect resident cells that expressed ACE2 receptor, and indirect factor involving cytokine storm induced hypercoagulable and hyperthrombotic state (Sweid *et al.*, 2020). Same mechanism involving direct cytopathic effects through ACE2 receptor expressed tissues, and indirect effects through cytokine storm induced damage have been ascribed for cardiovascular and nephrological manifestations of COVID-19.

New evidence emerging that also needs to be further studied is revealing that COVID-19 as respiratory illness might originate from vascular side of alveolus rather than epithelial side (Marini and Gattinoni, 2020), with evidence of widespread pathological findings in endothelial cells of pulmonary and renal blood vessels among patients who died from COVID-19. This increasing evidence of findings suggested COVID-19 as vasculotropic disease that infects blood vessels and cause endothelial damage instead of purely and primarily respiratory illness and thus, the potentiality to change the scientific world view about mechanism through which COVID-19 causes fatal outcomes and consequently new approach to development of therapy. There are also increasing anecdotal evidence from frontline medical professionals about patients presenting with symptoms of low oxygen saturation and shortness of breath but without abnormal lungs findings on CT scan and yet test positive to COVID-19. Such finding of well-preserved lung mechanics despite presence of hypoxemia among COVID-19 patients has also been observed and documented (Magro *et al.*, 2020), it also raised question as to whether the ARDS associated with SARS-CoV-2 is nonconforming with well-established mechanism of typical ARDS.

### Points of concern for research communities

The following points of concern stimulate many questions for research communities with respect to COVID-19 in Nigeria:

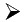
 The trends of the SARS-CoV-2 transmission and low virulency of the virus in Nigeria relative to the other regions of the world.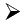 The reported rise in mass mysterious deaths in Northern part of Nigeria especially during the initial period of COVID-19 lockdown ordered by the Government.

With respect to the first point of concern, as above stated by 5^th^ May 2020 that correlate with 10 weeks period from the first index case, a total confirmed case of 2,950 and total of 98 deaths were recorded in Nigeria. Comparatively, COVID-19 moved from index cases to 161,807 (2,978), 143,626 (18,279), and 91,472 (5,806) confirmed cases (fatalities) within 10 weeks in US, Italy, and Iran respectively. Similar comparison that matched data and depicted corroborated findings in number of cases and case mortality index between Nigeria and other countries in the world was numerously pointed out by Nigerian authors in various Nigerian COVID-19 studies (Ohia et al., 2020).

The first question to this point of concern relates to whether the strain of SARS-CoV-2 ravaging Nigeria is relatively less pathogenic and virulent or otherwise not. There is evidence of SARS-CoV-2 recurrent mutation and emergence of genomic diversity in human samples of the virus (Dorp *et al.*, 2020). Mutation is the slight change in the viral genetic code to create a slightly different strain as it passes from one person to another as it reacts with evolutionary pressure from localized immune responses or drugs active against the virus. Mutation of SARS-CoV was demonstrated to make the virus either more virulent or less virulent. A study on SARS-CoV established that mutations in spike (S) protein and nonstructural protein 9 (NSP9) conferred the virus with additional virulence by increasing the recognition for the virus S protein to ACE2 receptor (Frieman *et al.*, 2012). The first study in the world to document the whole genomic sequence SARS-CoV-2 originated from Wuhan, China (Wu *et al.*, 2020), and the first African study to document SARS-CoV-2 genomic sequence originated from Nigeria (Happi *et al.*, 2020). Since from the first world genomic study, more than 500 genomic sequences of SARS-CoV-2 have been uploaded and still counting by scientists all over the globe into the global databank termed global initiative on sharing all influenza data (GISAID). The potentiality of SARS-CoV-2 genetic mutation to change the course of the viral pathogenicity and transmissibility have been variously detected (Tang *et al.*, 2020). However, some experts argued that mutations of viruses, including SARS-CoV-2, are inherently part of the viral life cycle and in the case of SARS-CoV-2 could seldom be to significant extent as to impact the course of the disease (Grubaugh *et al.*, 2020). Nevertheless, study of the continuing evolution and genomic diversity characterization of SARS-CoV-2 among Nigerian population with COVID-19 disease might confirm such claims or otherwise.

The second question to this point of concern involves whether it could be possible that the relative limited diagnostic testing capacity of SARS-CoV-2 in Nigeria is actually masking the true number of confirmed infected cases or not. As of 5^th^ May 2020, the total figure for people tested for COVID-19 in Nigeria since the beginning of the pandemic stood at 21,208 with the average number being tested in 24 hours stood at 1,696 (NCDC, 2020). Despite the fact that massive testing for diagnosis has been identified as keystone for the control of the pandemic, an ideal massive testing capacity is limited all over the world including in the developed countries (Amanat *et al.*, 2020). But still Nigeria’s testing capacity is comparatively inadequate and less extensive. For instance, the Center for Disease Control) in US weekly report (April 19 – April 25, 2020) revealed the number of specimens tested within that week period as 701,913 which is equivalent to an average of more than hundred thousand diagnostic test per day. This is also substantiated by some of the studies conducted in Nigeria that also attributed the relative low number of confirmed COVID-19 cases and slow infectivity rate of the virus to the extent of access to or availability of testing facilities (Bamidele and Daniel, 2020). This has also shown that despite all efforts to increase testing, Nigeria has to beep up to meet global coverage in terms of testing. As such, the relative disproportionate figures of COVID-19 from Nigeria could only be subjected to definitive proof or otherwise by relative increase in the country’s diagnostic testing capacity or mass testing survey of the population.

The third question to this point of concern relates to whether it could be possible that the number of asymptomatic COVID-19 cases in Nigeria is so significant that many might have had the disease asymptomatically unreported and recovered. A preliminary data of 21 reports analysis has revealed that between 5% and 80% of SARS-CoV-2 positive patients are asymptomatic (Heneghan *et al.*, 2020) and this is not surprising as this is virtually true with all infectious diseases, that the number of those infected with the disease exceeds the number of those presenting with complaint of the disease because immune system might have overcome the disease in many people undetected (Machado *et al.*, 2004). It might be possible that significant proportion of Nigeria’s population might have had strain of SARS-CoV-2 asymptomatically unreported and recovered, signifying herd immunity. Only that for herd immunity otherwise called community immunity to develop, about 80% of the population must have gotten infected (Naafs, 2018). Is this the case for the population of Nigeria? Perhaps serological studies of SARS-CoV-2 antibody test among Nigerian general population could be able to generate potential information on whether Nigerians might have had COVID-19 and been immune to it, hence becoming undetected. Such population-based serological studies for SARS-CoV-2 specific antibodies have been carried out in many parts of the world (Sood *et al.*, 2020). A related and equally important question to pursue is how robust is the immunity developed following the COVID-19 infection in Nigeria. Whether it could actually protect one from developing the disease, or does it just reduce the severity of symptoms? How long the immunity does lasts, for a lifetime or is it just a concomitant immunity?

As relate to the second point of concern, it was noted that within some few weeks of lockdown in Kano - arguably most densely populated city in Northern part of Nigeria, 150 people died in three days and a total of about 640 people were reported dead within two weeks in the same community, a figure considered abnormally high by the authority and people including grave diggers (Daily Trust, 2020a). The reason for such death spark spurious reactions from government and people as to whether such deaths were COVID-19 related or not. As such an important question to this point of concern here is whether such mysterious deaths were due to COVID-19 or not. Preliminary data involving a verbal autopsy interview with the families of the deceased in Kano which was carried out by the Presidential Technical Team in Kano State suggested that COVID-19 was most likely the cause of those deaths, as most of the responses generated, reported COVID-19 typical symptoms, like fever, shortness of breath, by the deceased before their death (Daily Trust, 2020b). However, a retrospective autopsy study into cases of such death could verify such claims.

Another question on the cause of such mass mysterious deaths is whether the unconfirmed COVID-19 had compounded and complicated co-morbidity conditions that could be responsible for their death or not. It is known that the most solid predictor of COVID-19 severity and mortality are older age and other multiple or specific co-morbidities such as hypertension and diabetes (Emami *et al.*, 2020). Preliminary data suggested that the mysterious deaths involved advanced aged group or people with underlining health conditions (Daily Trust, 2020c). Additionally, it was argued whether the cause of such mass mysterious deaths was related to sudden change to sedentary lifestyle imposed by the lockdown order or not. It is documented that maintaining regular physical activity during lockdown, self-isolation, quarantine or physical distancing is important for prevention and attenuation of negative health consequences such as depression, type 2 diabetes, cancer, venous thrombosis and thromboembolism (Jakobsson *et al.*, 2020). Prolonged sitting, sedentary lifestyle and inadequate moderate to vigorous intensity physical activity have been reported to be associated with cardiovascular disease (CVD) mortality risk in adults (Stamatakis *et al.*, 2019). For this reason, WHO demonstrated practical sessions of home-based exercise programs that could be useful to stay physically active during COVID-19 induced quarantine (WHO, 2020b). This should be a guide to lockdown situation that people are encouraged to follow guidelines by WHO on how to cope and mitigate the negative health consequences of the lockdown.

Another question is the probability of whether lack of fully functional hospital services due to the lack of personal protective equipment among healthcare workers is actually causing or contributing to such mass mysterious deaths or not. Safety of healthcare workers who are the mainstay for combating against the disease have been on the limelight since the first outbreak of the disease, as many were reported to have been infected in the course of discharging their duties (Jin *et al.*, 2020). About 113 healthcare workers were infected with COVID-19 in Nigeria as of 1^st^ May, 2020 (Punch, 2020), and this has been attributed to anecdotal lack of PPEs prompting most healthcare workers to be out of their duty post especially at the tertiary and private hospitals.

In another development, Kano has recently experienced a relative sharp decline in number of COVID-19 cases and such mysterious deaths’ report, and on 2^nd^ July, 2020 the state government totally removed the lockdown. But considering the fact that COVID-19 pandemic is not yet over; the Nigerian low vulnerability index for infectious disease of 0.27 out of a maximum of 1.0 (Ibrahim and Olasinde, 2020) indicating potential catastrophic consequences if impending key questions are not being scientifically answered and addressed; and for posterity sake (Paintsil, 2020). Such mysterious death issue should never be swept under the carpet and those questions should never be left unraveled, not only in Kano but also in all parts of northern Nigeria where such mysterious deaths have been reported.

### Intelligent approaches to COVID-19

With the outbreak of COVID-19, plethora of technology based computing intelligent approaches have been used as non-clinical techniques and proven very powerful tools towards impacting the containment of the pandemic. These intelligent approaches include both the advance and traditional, with advance computational intelligent (CI) approaches such as biosensors, artificial intelligence, fuzzy logic decision systems, tele-based healthcare and data mining models been the most prominent in COVID-19 pandemic.

Biosensors possess the capability to detect and convert specific biological response into signals that can be quickly translated, as such nanomaterials enabled biosensors have been found to offer a very fast and early diagnosis of COVID-19 cases (Srivastava *et al.*, 2021). Artificial intelligence using artificial neural networks and machine learning systems have been statistically assessed and found to be valuable in predicting the results of molecular diagnostic test for SARS-COV-2 (Langer *et al.*, 2020). Numerous artificial intelligence approaches were recommended to offer the best remedy for maximizing safety and preventing the spread of COVID-19 (Adly *et al.*, 2020). Fuzzy logic-based decision systems have been proven useful for prediction and forecasting of COVID-19 pandemic, typical example is neuro-fuzzy inference system which has been proposed as an ideal COVID-19 forecasting model (Al-qaness *et al.*, 2020), which assist in guiding authorities in the development of better public health strategies and in taking productive decisions (Shinde *et al.*, 2020). Tele-based healthcare has been systematically reviewed and found to facilitate the provision of health services during COVID-19 outbreak (Monaghesh and Hajizadeh, 2020). Data mining models developed to test for the prediction of COVID-19 infected patients’ recovery, were found to be best in terms accuracy with overall of 99.85 percent (Muhammad *et al.*, 2020).

Traditional intelligent approaches such as SIR (susceptible-infective-recovered) model have been used to analyze and predict the spread of COVID-19, as well as forecast COVID-19 pandemic trends (De lima *et al.*, 2020). SIR model has been used in predicting the COVID-19 19 pandemic in Nigeria, and the results obtained when compared with that of four other countries namely South Africa, Italy, US and China, found to be less in Nigeria (Udanor and Emeh, 2020). This finding was also corroborated using another traditional intelligent approach termed Bayesian method aimed to estimate the transmissibility reproduction number of COVID-19 in Nigeria at different time intervals, with the result found illustrating the relative lower transmission rate in Nigeria (Adegboye *et al.*, 2020).

## Conclusion

Pathogenesis of COVID-19 remains an emerging knowledge and there are many important research questions that need to be scientifically answered for a successful containment of COVID-19 in Nigeria. Fighting against COVID-19 pandemic should not be seen or regarded as just the work of medical or health scientists as all members of intellectual research communities could have a potential role to play to collectively combat the menace of COVID-19 pandemic.

**List of Abbreviations:**
(COVID-19): Coronavirus Disease 2019
(SARS-CoV-2): Severe Acute Respiratory Syndrome Coronavirus 2
(UNCTAD): United Nations Conference on Trade and Development Agency
(GDP): Growth Domestic Product
(WHO): World Health Organization
(WHO ICTRP): World Health Organization’s International Clinical Trials Registry Platform
(2019-nCoV): 2019-Novel Coronavirus
(CSG): Coronavirus Study Group
(ICTV): International Committee on Taxonomy of Viruses
(PHEIC): Public Health Emergency of International Concern
(NCDC): Nigeria Centre for Disease Control
(CoVs): Coronaviruses
(RNA): Ribonucleic Acid
(US): United State
(SARS): Severe Acute Respiratory Syndrome
(MERS): Middle East respiratory syndrome
(ssRNA): Single-Stranded RNA
(ORFs): Open Reading Frames
(pp1a): Polyprotein 1a
(pp1b): Polyprotein 1b
(NSPs): Non-Structure Proteins
(ACE2): Angiotensin-Converting Enzyme 2
(gRNA): Genome RNA
(RTC): Replication-Transcription Complex
(TIR): Toll/Interleukin-1 Receptor
(TIRAP): TIR-Domain-Containing Adaptor Protein
(TRIF): TIR-Domain-Containing Adapter-Inducing Interferon-β
(MAVS): Mitochondrial Antiviral-Signaling Protein
(STING): Stimulator of Interferon Genes
(NFκB): Nuclear Factor kappa B
(IRF3): Interferon Regulatory Factor 3
(SPO_2_): Saturated Partial Pressure of Oxygen
(CS): Cytokine Storm
(ARDS): Acute Respiratory Distress Syndrome
(IL): Interleukin
(IFN): Interferon
(TNF): Tumor Necrosis Factor
(CSF): Colony Stimulating Factor
(GF): Growth Factor
(IFN-γ): Interferon Gamma
(TNF-α): Tumor Necrosis Factor Alpha
(MCSF): Macrophage Colony-Stimulating Factor
(MCP-1): Monocyte Chemoattractant Protein 1
(HGF): Hepatocyte Growth Factor
(CNS): Central Nervous System
(CT): Computed Tomography
(GISAID): Global Initiative on Sharing All Influenza Data
(CDC): Center for Disease Control
(CVD): Cardiovascular Disease
